# Effects of water level fluctuation on thermal stratification in a typical tributary bay of Three Gorges Reservoir, China

**DOI:** 10.7717/peerj.6925

**Published:** 2019-05-15

**Authors:** Juxiang Jin, Scott A. Wells, Defu Liu, Guolu Yang, Senlin Zhu, Jun Ma, Zhengjian Yang

**Affiliations:** 1 School of Water Resources and Hydropower Engineering, Wuhan University, Wuhan, Hubei, China; 2 Department of Civil and Environmental Engineering, Portland State University, Portland, OR, USA; 3 Hubei Key Laboratory of Ecological Restoration of River-Lakes and Algal Utilization, Hubei University of Technology, Wuhan, Hubei, China; 4 State Key Laboratory of Hydrology-Water Resources and Hydraulic Engineering, Nanjing Hydraulic Research Institute, Nanjing, China

**Keywords:** Thermal stratification, CE-QUAL-W2, Three Gorges Reservoir, Xiangxi River, Water level fluctuation

## Abstract

Xiangxi River is a typical tributary of Three Gorges Reservoir (TGR) in China. Based on field observations in 2010, thermal stratification was significant in most months of the year. Through field data analysis and numerical simulations, the seasonal and spatial variation of thermal stratification as related to the impact of the operation of TGR were investigated. Thermal stratification was most pronounced from April to September in the Xiangxi River tributary. Air temperature (AT) and water level (WL) were the two dominant variables impacting thermal stratification. AT affected the surface water temperature promoting the formation of thermal stratification, and high WLs in TGR deepened the thermocline depth and thermocline bottom depth. These results provide a preliminary description of the seasonal variation and spatial distribution of thermal stratification, which is important for better understanding how thermal stratification affects algae blooms in Xiangxi River.

## Introduction

Reservoirs are constructed for multiple purposes, such as hydropower production, flood control, water supply, and commercial fisheries. While reservoirs provide benefits for society, they can also have negative impacts on ecosystems. For example, serious algal blooms have occurred in many tributaries of Three Gorges Reservoir (TGR) in China since its impoundment (Liu et al., 2012; Zhuan-xi et al., 2007). These blooms were not only influenced by water temperature, solar radiation, and nutrients, but also by hydrodynamics and thermal stratification (Wu, Liu & Hsieh, 2004; Tufford & McKellar, 1999). Since the surface water temperature is affected by incoming long wave radiation, evaporation, heat conduction and solar radiation, thermal stratification is common in many reservoirs and deep lakes (Ma et al., 2008; Kim & Kim, 2006; Boland & Padovan, 2002). Stratification can provide a stable habitat for the growth of phytoplankton and result in algae bloom if other conditions, such as nutrients, are favourable. To control the water quality in the tributaries of TGR and to optimize its operational objectives, it is necessary to study the hydrodynamic characteristics of thermal stratification in the tributaries influenced by the operation of TGR.

Thermal stratification in reservoirs is affected not only by meteorological conditions (Owens & Effler, 1989; Effler et al., 1986), but also by the operation of the reservoirs (Milstein & Zoran, 2001; Han et al., 2000). Xiangxi River is the nearest tributary to the TGR dam (32 km upstream), and is more directly influenced by the TGR operation (primarily water level fluctuations (WLFs)) than the other tributaries further upstream. The maximum WLF between the flood and dry seasons can reach almost 30 m, which can significantly affect the hydrodynamics of the tributaries. Although many studies have addressed the hydrodynamics of Xiangxi River (Ma et al., 2015; Yang, 2014; Jiang, Dai & Liu, 2013; Ji et al., 2010a, 2010b), research is very limited on the variation of thermal stratification and the interaction between thermal stratification and hydrodynamic process as influenced by the operation of the TGR.

The goal of the present study is to develop an understanding of how thermal stratification is affected by WLFs through field observations and numerical simulations. It is hoped that this study will contribute to building a foundation for studying the interaction between thermal stratification and algae blooms in the Xiangxi River.

## Region of Interest and Methods

### Region of interest

**Figure 1:**
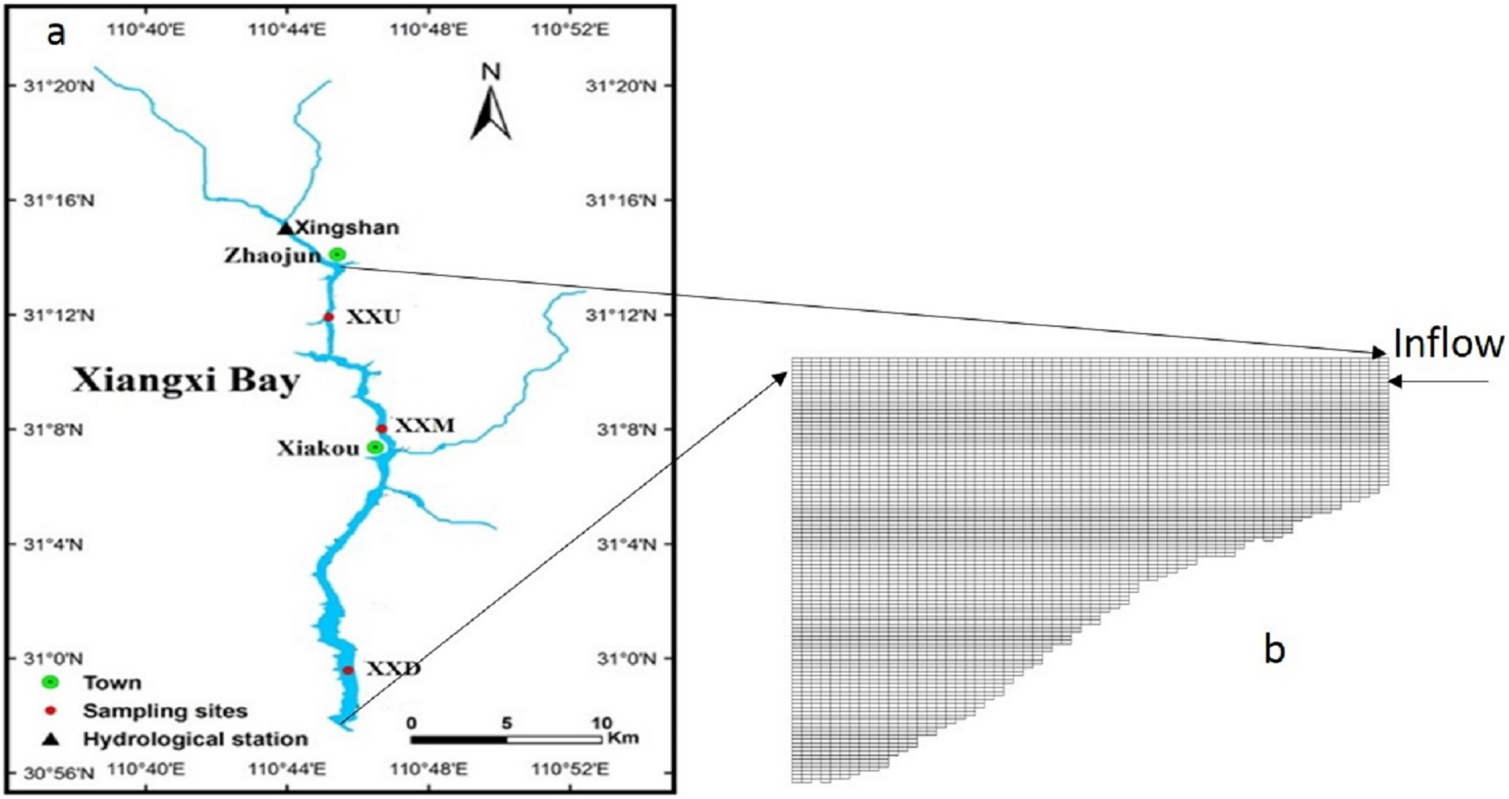
Location of the Xiangxi River. (A) Three sampling sites are from upstream to downstream in Xiangxi River: upstream (XXU), middle (XXM), and downstream (XXD); (B) grid used in the CE-QUAL-W2 model of the Xiangxi River showing longitudinal segments and vertical layers.

Xiangxi River is located in Hubei Province, China. The length of Xiangxi River is 94 km starting from Shennongjia Forest Region to Yangtze River, and the catchment area is approximately 3,095 km^2^. Due to the operation of TGR, the 30 m WLF between the flood and dry seasons results in large variations on the backwater area of the Xiangxi River. The water from the Yangtze River intrudes into the Xiangxi River tributary when the TGR water level (WL) rise, and the maximum backwater area can extend to almost 40 km when the TGR WL reaches 175 m. This backwater area is referred to as Xiangxi Bay (XXB, Liu et al., 2012; see Fig. 1A). XXB is the focus of this study.

### Measured data

**Figure 2:**
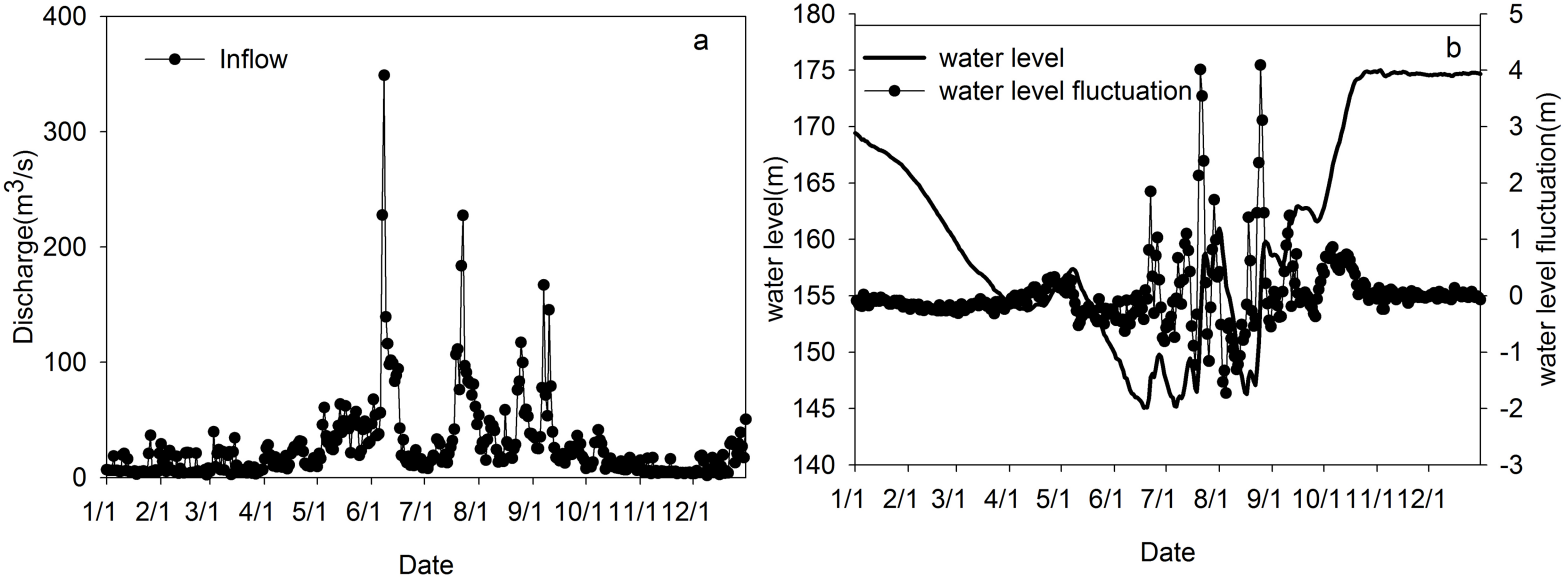
Hydrological conditions in 2010. (A) Upstream inflow; (B) the water level and daily water level fluctuations at the estuary of the Xiangxi River.

Inflow discharge data was measured in 2010 from a hydrological station at Xingshan (Fig. 2A). WL data at the most downstream point of Xiangxi River at the intersection with the TGR for 2010 was obtained from the China Three Gorges Corporation (see Fig. 2B). As shown in Fig. 2A, the average inflow was 26.8 m^3^/s, with a maximum value of 348.8 m^3^/s on June 8 and a minimum value of 1.6 m^3^/s on December 9, 2010. From Fig. 2B, the high WL of 175 m was reached in late October and the low WL of 145 m occurred in mid-June. Based on the daily WL data, we calculated the daily WLF by comparing it with the value from the previous day. The daily WLF varied sharply during the flood season, with values ranging from 4.09 m on August 25 to −1.73 m on August 5.

Water temperature and the corresponding depth were measured using a Hydrolab DS5X multi-probe sonde (Hach, Loveland, CO, USA) with a vertical resolution of one m. Vertical profile measurements were taken weekly at sites XXU and XXD, and daily at site XXM.

**Figure 3:**
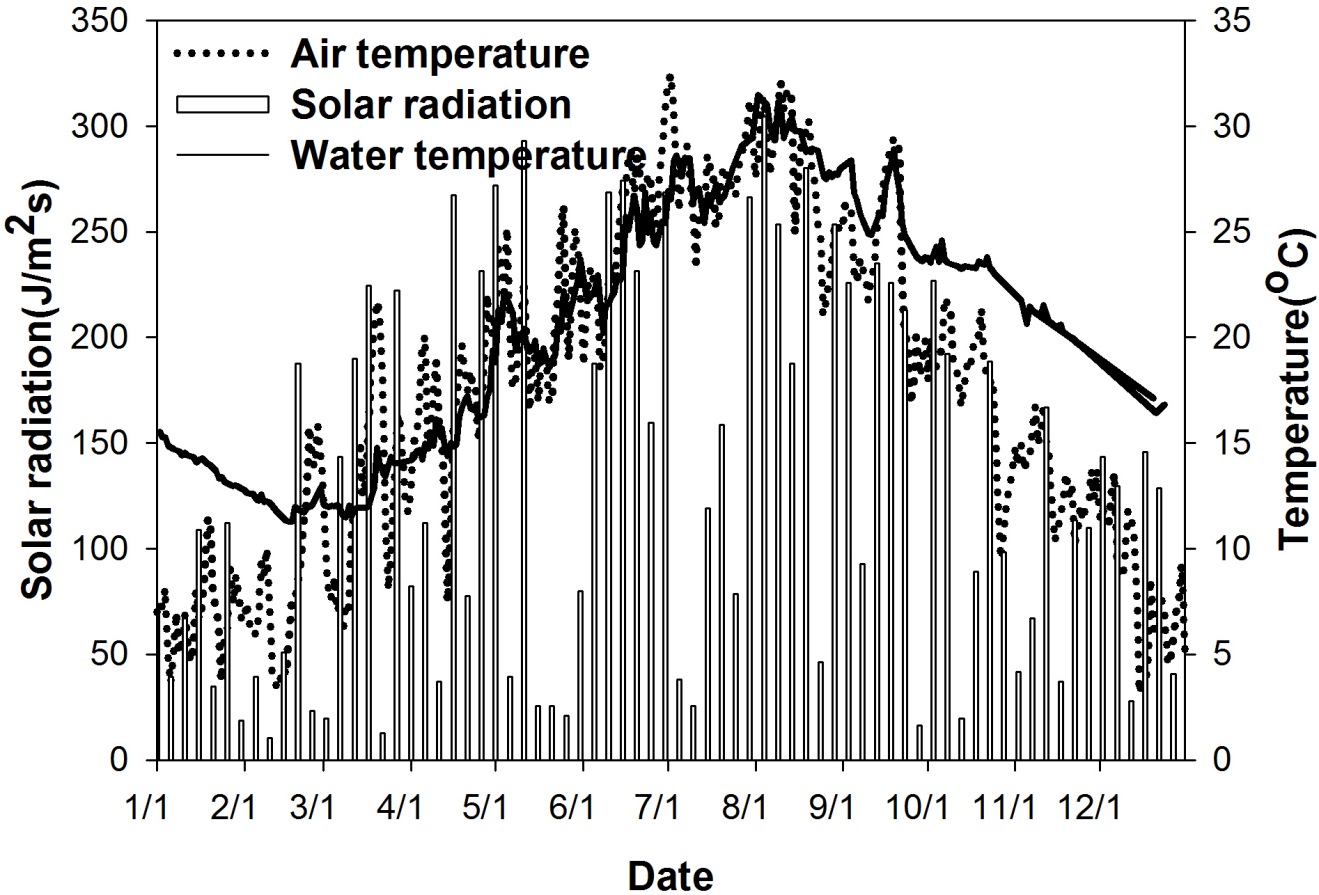
Measured air temperature, solar radiation, and surface water temperature in the Xiangxi River in 2010. This graph displays seasonal variations in meteorological conditions (air temperature, solar radiation) and surface water temperature.

Daily meteorological data were collected from the Xingshan Hydrological Station. The data show that the average AT was 17.4 °C, with a maximum of 32.4 °C on July 2 and a minimum of 3.2 °C on December 15. The average solar radiation was 127.5 J/m^2^s with a range from 5.8 to 304.4 J/m^2^s. The average surface water temperature was 20.1 °C, with a maximum of 31.0 °C on August 2 and a minimum of 11.3 °C on February 17 at site XXM. The surface water temperature, AT and short wave solar radiation in 2010 is shown in Fig. 3. AT from April to September correlated well with surface temperature with a correlation coefficient of 0.86.

## Ce-Qual-W2 Model

### Model description

The CE-QUAL-W2 (Cole & Wells, 2017) model is a vertical, 2D, laterally averaged hydrodynamic and water quality model that has been applied successfully in many stratified waterbodies (Sadeghian et al., 2018; Zhang, Sun & Johnson, 2015; Noori et al., 2015; Deus et al., 2013; Thomson & Fine, 2003). Because the model assumes lateral homogeneity, it is well-suited for relatively long and narrow waterbodies, which is the case for the XXB (Ma et al., 2015). Thus the CE-QUAL-W2 model was selected to simulate the hydrodynamic characteristics of XXB.

### Simulation conditions and calibration

The model grid for the Xiangxi River was divided into 64 longitudinal segments of 500 m in length, and 109 vertical layers of one m in thickness; the entire water column was configured for a total of 4,385 cells (see Fig. 1B). The maximum depth was 100 m at the most downstream segment (the mainstem Yangtze River), while the depth in the upstream sections was only a few meters. To study the thermal stratification and its seasonal variation, field observation data for a complete year were acquired. Based on available field measurements, the simulated period was from January 1 to December 31 in 2010. The boundary conditions (upstream flow and downstream head) used for the simulation are shown in Fig. 2.

Calibration is the process of adjusting appropriate model parameters by matching the simulation results with the observed data. When the model can reproduce the observed results well, then it can be applied to simulate different but similar scenarios. Hydrodynamic conditions can be affected by several model parameters, such as the longitudinal eddy viscosity, longitudinal eddy diffusivity by influencing the temperature and hence density, Manning’s roughness coefficient, and the wind-sheltering coefficient. Little adjustment of model parameters or calibration was performed since most of the parameters used in the model were assigned default values. The wind sheltering coefficient was adjusted to 0.9 for Xiangxi River according to a previous study (Ma et al., 2015). This coefficient corrects the wind on the Xiangxi River from the measurement location by reducing the wind 10% throughout the entire waterbody. The model coefficients affecting hydrodynamics used in the study are shown in Table 1 and are largely default model coefficients.

**Table 1:** The coefficients used in the model for XXB.

Coefficient	Value
Longitudinal eddy viscosity	1.0 m^2^/s
Longitudinal eddy diffusivity	1.0 m^2^/s
Manning’s roughness coefficient	0.04 s/m^1/3^
Wind sheltering coefficient	0.9

### Data analysis

The thermocline refers to the profile of maximum decrease rate of temperature in the metalimnion, and methods for estimating the thermocline from profile data including threshold of temperature and density or a critical gradient (Chung & Gu, 2009). In this study, the temperature-gradient threshold was used to calculate the thermocline parameters (Zhang et al., 2014). The uniform criterion of 0.2 °C/m was selected to determine the thermocline according to previous research on Xiangxi River (Liu et al., 2012). The thermocline depth (TD) and thermocline bottom depth (TB) were used as the scale of thermal stratification. The TD was defined as the depth of the upper most part of the thermocline, and the TB was defined as the depth of the lower most part of the thermocline (Liu et al., 2019), the thermocline thickness was the distance between the TD and the TB. Correlation analysis was performed using R software.

## Results and Discussion

### Thermal stratification in Xiangxi River

**Figure 4:**
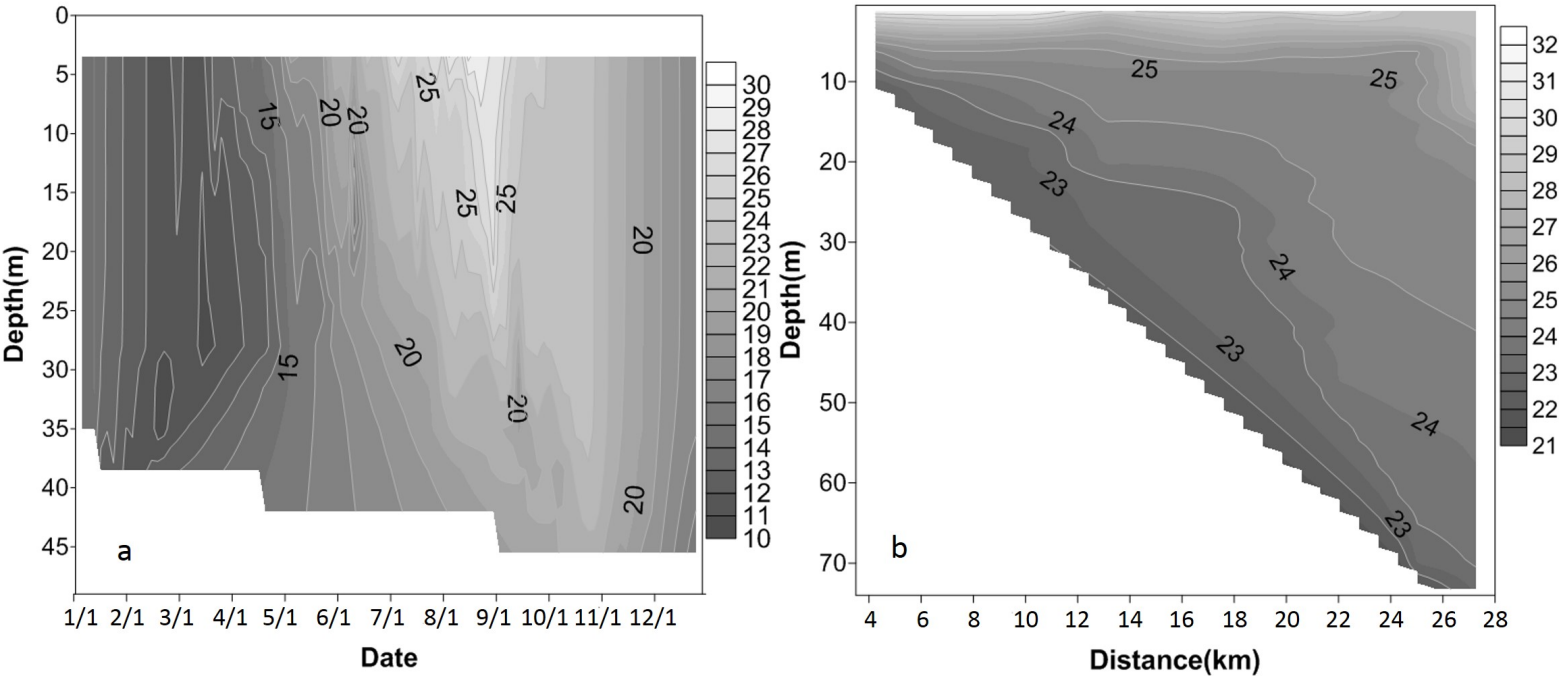
Vertical profile of water temperature measured in Xiangxi River in 2010. (A) Contour of water temperature measured at site XXM in 2010; (B) contour of water temperature in Xiangxi River on August 9, 2010, the flow direction is from left to right.

The seasonal variation in the water temperature at site XXM was analysed using data measured in 2010. As shown in Fig. 4A, the seasonal temperature variation was significant in Xiangxi River. At the surface layer, the minimum water temperature occurred between February and March, then increasing rapidly from April to its maximum value of approximately 31 °C in August. This seasonal variation of the water temperature of the surface layer was also seen in the variation of AT and solar radiation (see Fig. 3). Water temperature decreased from September to December. As the water depth increased, the variation in water temperature significantly trailed the AT variation. The maximum water temperature at different vertical elevations was reached at different times. For example, the maximum water temperature at the surface layer occurred in August, while the maximum water temperature at a depth of 35 m occurred in mid-October.

Figure 4 shows the stratification and destratification processes at site XXM. The water temperature rapidly increased at water depths between 0 and 15 m from 11 to 31 °C between February and August, with an increase of temperature at a rate of 0.1 °C/day. The rate of temperature increase rate was approximately 0.05 °C/day during the same period at a depth of 35 m. However, the maximum rate of increase of water temperature in the deeper layers could be higher than that of the surface layers. For example, the maximum value of the temperature increase rate was 0.23 °C/day (from May 21 to July 14) at a depth of 15 m, while the maximum rate of 0.51 °C/day (from April 16 to May 6) was found at a depth of 25 m. At a depth of 35 m, the increase rate was 1.28 °C/day (from February 1 to 10). This result may, however, be related to the hydrological conditions of Xiangxi River, with large temperature changes originating from inflow currents.

The surface water temperature began to rapidly decrease beginning in September (Fig. 3), and the epilimnion deepened following the reduced surface water temperature. The epilimnion reached a depth of 30 m in September, and the depth increased to 40 m in November. An unstable water temperature structure was observed from February to March, which triggered strong vertical mixing. The surface water temperature reached a minimum value in February. Stratification was therefore weak, and the waterbody was unstable, leading to strong vertical mixing.

The spatial variation in the water temperature of Xiangxi River was evaluated using data from August 9, 2010. As shown in Fig. 4B, there were thermal layers in Xiangxi River, and the thickness of each layer changed longitudinally from upstream to downstream.

### Simulation of thermal stratification

**Figure 5:**
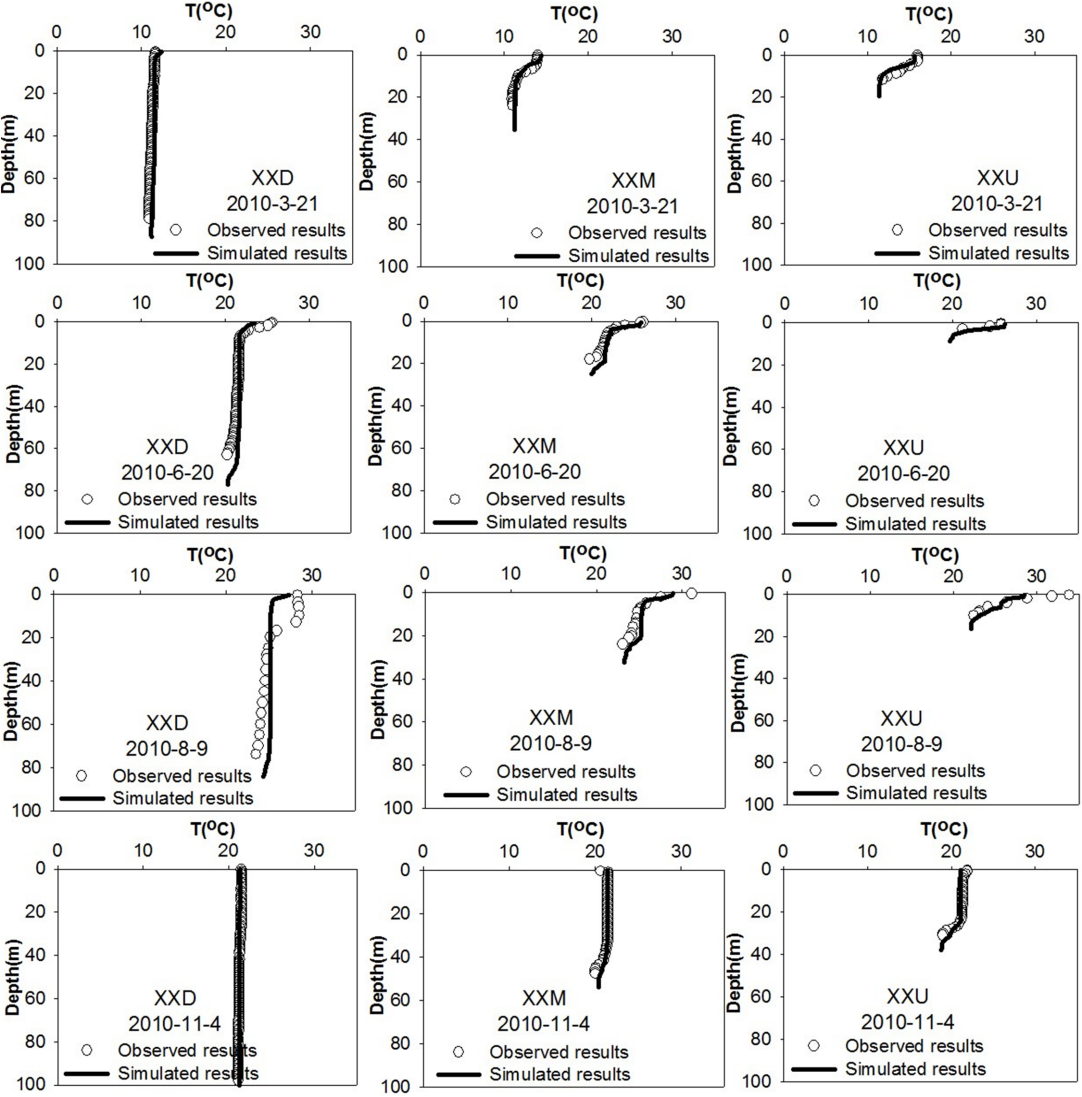
Calibration assessments of the vertical temperature profiles. These graphs display the observed and simulated vertical profiles of the water temperature at sites XXD, XXM and XXU, from downstream to upstream in the Xiangxi River.

Since thermal stratification was observed between April and September, the numerical model results were compared with data measured during this period. As shown in Fig. 5, the model not only reproduced the temperature profile but also the formation and duration of the thermal stratification. The simulated thermocline agreed well with the field-measured data from the downstream to the upstream, with an error of generally less than 1 °C. The simulated model results were consistent with the conclusions drawn by the analysis of measured data.

Thermal stratification was not evident in the downstream section of the XXB in late March, but stratification was significant in the middle and upstream sections during this period. In June, thermal stratification developed at a depth of 10 m in the downstream section of the XXB. The depth of the thermocline increased following the increased surface water temperature such that by August it had increased to a depth between 13 and 20 m. The water temperature difference between the surface water and the bottom was approximately 3 °C in August but completely disappeared by November at the downstream station, which indicated that the duration of thermal stratification in the middle and upstream portions of the XXB was longer than that in the downstream portion, reflecting the influence of the Yangtze River.

### The impact of water level fluctuation on thermal stratification

The operation of TGR can cause WLFs which influence the hydrodynamic processes and the thermal regime in the Xiangxi River. Based on hydrological data from 2010, we found that WL significantly varied from June to October. During this period, only consecutive daily WLFs greater than 0.2 m/day were considered (see Table 2). The maximum total WLF was −14.03 m, which occurred from August 2 to August 16. The maximum mean daily WLF was 2.46 m/day, which occurred from July 20 to July 24. This result indicated that the WL rose and fell alternately from late June to mid-August. Then, the WL rose gradually, starting from late August, and reached a maximum elevation in late October.

**Table 2:** Statistics of water level variation in Xiangxi River in 2010.

Date	Initial and terminal water level (m)	Total water level variation (m)	Mean daily water level fluctuation (m/day)	Air temperature variation (°C)
June 21–June 23	145.9–148.09	2.19	1	27.0–27.8 (27.3*)
June 28–July 4	149.59–146.07	−3.52	−0.53	27.7–32.4 (30.1*)
July 11–July 15	146.2–149.45	3.25	0.71	23.5–28.6 (25.9*)
July 16–July 19	148.91–146.46	−2.45	−0.75	25.2–27.9 (26.7*)
July 20–July 24	148.59–158.76	10.17	2.46	26.8–28.1 (27.5*)
July 25–July 27	158.08–156.71	−1.37	−0.68	26.7–28.6 (27.8*)
July 28–August 1	157.53–160.97	3.44	0.85	27.6–31.2 (29.5*)
August 2–August 16	160.46–146.43	−14.03	−0.97	25.0–32.1 (29.8*)
August 23–August 28	148.54–159.79	11.25	2.12	21.1–25.4 (23.2*)
September 8–September 11	158.2–161.62	3.4	0.96	21.8–23.3 (22.7*)
September 29–October 24	161.96–174.7	12.74	0.57	16.7–21.9 (19.1*)

**Note:**

* Mean air temperature. Only continuous daily water level fluctuation greater than 0.2 m/day and a total water level variation greater than one m were taken into account in this statistics.

### Thermal stratification structure variation

**Figure 6:**
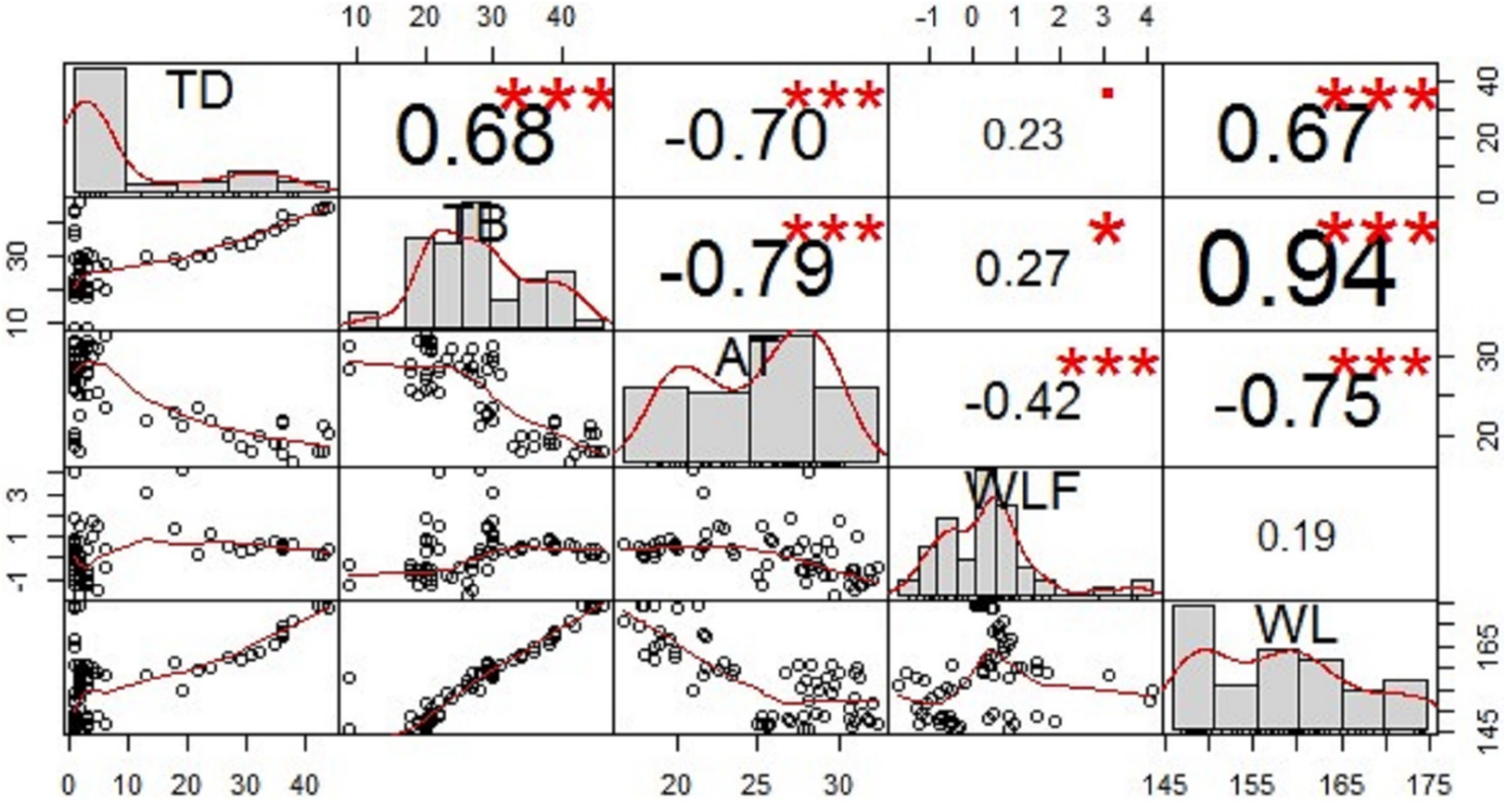
Correlation relationships of the thermocline depth (TD), thermocline bottom depth (TB), air temperature (AT), daily water level fluctuation (daily WLF), and water level (WL). This graph displays the correlation coefficients and distributions of the thermocline parameters (TD, TB) and the impact factors (AT, WLF, WL); the number of asterisks indicate the strength of the correlation, and the numerical value represents the correlation coefficient between the corresponding variables.

The analysis of the correlation between TD and TB and the impact factors of AT, daily WLF, WL reveals that AT and WL had a significant effect on the thermocline parameters (see Fig. 6). AT was negatively correlated with TD and TB. The correlation coefficient between AT and TD and TB were −0.70 and −0.79, respectively, indicating that both the TD and the TB deepened with decreasing AT. The correlation between the daily WLF and the TDs was not significant during this period. The WL was positively correlated with the TDs. The correlation coefficient between the WL and TD and TB were 0.67 and 0.94, respectively. It indicates that WL variation had a strong effect on the thermal stratification structure.

**Figure 7:**
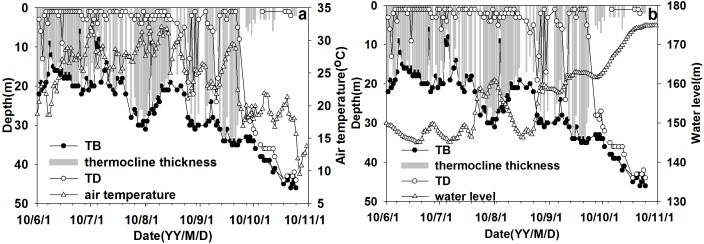
The variation process of thermocline parameters with air temperature\water level (WL) from June to October at site XXM. (A) The variation process of thermocline depth (TD)\thermocline bottom depth (TB)\thermocline thickness with air temperature (AT); (B) the variation process of thermocline depth (TD)\thermocline bottom depth (TB)\thermocline thickness with water level (WL).

The variations of the TD, the TB, the AT and the WL at XXM were evaluated from June to October 2010. As showed in Fig. 7, it was found that the TD was zero to five m with slight changes from June to late September, then below 25 m in October. The TB gradually increased from around 20 m on August 15 to approximately 45 m on October 15. The thermocline thickness varied from 1 to 44 m with a mean value of 18 m. The mean value of thermocline thickness was 19 m from June to late September, then decreased to three m in October. The WL significantly changed from late September to October, demonstrating that both the TD and TB deepened following increasing WL (see Fig. 7B). Hence, water level variations can affect the thermal stratification structure significantly.

### The effect of various water levels on thermal stratification

To evaluate the effect of WL on thermal stratification, the CE-QUAL-W2 model was used to simulate different hypothetical situations. Since the WL and thermal stratification varied significantly from June to October in 2010, simulations were performed for the period between June 1st and October 31st. The range of WL was from 145.1 to 174.9 m, AT from 9.5 to 32.5 °C with a mean value of 24.4 °C, the inflow discharge from 6.1 to 348.8 m^3^/s, and the inflow temperature from 6.8 to 21.5 °C during this period. Hypothetical situations were designed in which WL was the only variable in each design condition. The baseline scenario used the measured field data as boundary conditions. The other two scenarios varied the WLF by ±5%. The other meteorological and hydrological conditions used the measured boundary conditions (see Table 3).

**Table 3:** Boundary conditions of hypothetical situations from June to October in XXB in 2010.

Experimental case	Water level (m)	Air temperature (°C)	Inflow discharge (m^3^/s)	Inflow temperature (°C)	Mainstream temperature (°C)
Baseline	145.1–174.9	9.5–32.5	6.1–348.8	6.8–21.5	20.7–27.5
Water level+5%	152.4–183.6	9.5–32.5	6.1–348.8	6.8–21.5	20.7–27.5
Water level−5%	137.8–166.2	9.5–32.5	6.1–348.8	6.8–21.5	20.7–27.5

**Figure 8:**
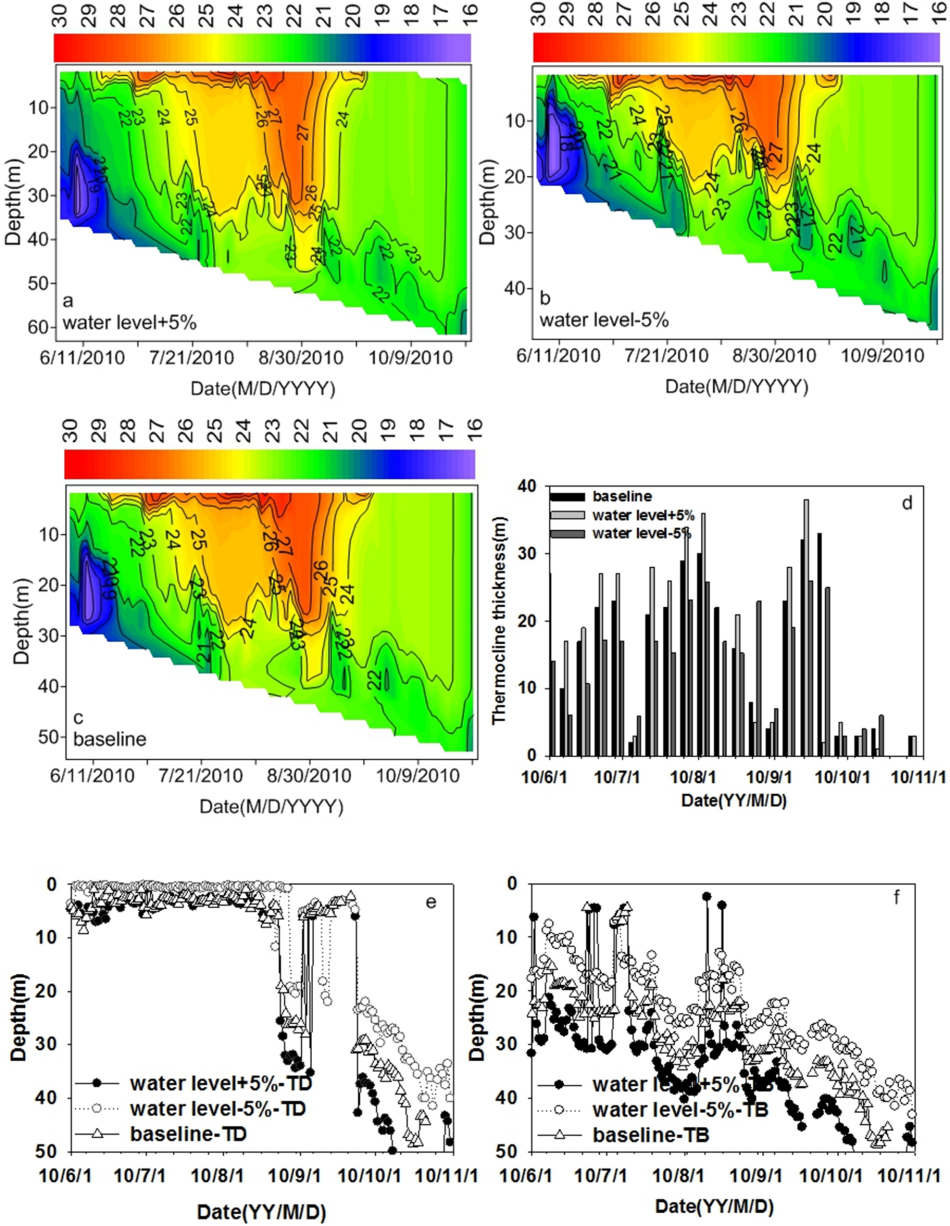
The effect of water level variation on thermal stratification at site XXM. (A) Water level increases by 5%; (B) water level decreases by 5%; (C) baseline; (D) thermocline thickness variation under different water levels; (E) thermocline depth variation under different water levels; (F) thermocline bottom depth variation under different water levels.

Variations in WLs affected the thermal stratification structure in Xiangxi River (see Fig. 8). The thermocline thickness increased with the increasing WL from June to late September, with little variation in October (see Fig. 8D). Both the TD and the TB increased when the WL increased by 5%, and the TD and the TB decreased when the WL decreased by 5% compared to the baseline simulation (see Figs. 8E and 8F). Hence, WL variation can affect the TD and the TB. As the WL rises, the TD and the TB were deepened. Nowlin et al. (2004) found reservoir stratification was more sensitive to WLFs than a natural lake. Naselli-Flores & Barone (2005) found that different hydrological years may result in different patterns of thermal stratification in summer. Wang et al. (2012) found that a low WL resulted in a higher surface temperature due to a decrease of impounded cold water. These studies indicated that WL variation can affect the thermal stratification, which is consistent with the findings of the current investigation.

## Conclusions

The process of thermal stratification resulting from the operation of TGR was studied using both field data analyses and numerical simulations for the period from June to October 2010. Analysis of the field observations in 2010 indicated that thermal stratification was significant during the summer months. Thermal stratification was evaluated using the thermocline upper and lower depth. The results indicated that WL and AT were two main factors which correlated with the thermal stratification. WL was positively correlated with the TDs, and the correlation coefficient between the TB and the WL was 0.94, indicating that WL variation can affect the thermal stratification vertical structure. AT was negatively correlated with the TD and the TB implying that the thermocline deepened following a decrease in AT. The correlation with air temperature is really a surrogate for correlation with surface heat transfer since air temperature directly affects long-wave radiation, evaporative, and conductive heat fluxes. Also, the air temperature is responding to the same meteorological forcing as the water body and hence this approximates the impact of all the atmospheric forcing at the air-water interface.

The CE-QUAL-W2 model was developed and calibrated using field observations, and results showed that it could satisfactorily reproduce the temporal and spatial variation of water temperature in Xiangxi River. The results of the numerical simulations indicated that the WLFs can affect the thermal stratification structure. The model showed that increasing WLFs were accompanied by the deepening of the TD and the TB, agreeing with the results of the field data analysis.

The formation of thermal stratification was usually followed by algae blooms in the spring. Since the thermal stratification structure can be affected by the WL variation under the operation of TGR, water level variation may be one of the tools that could be used to manage the growth of phytoplankton. The effect of variations in the thermal stratification structure on algae blooms requires further study.

## Supplemental Information

10.7717/peerj.6925/supp-1 Supplemental Information 1 Raw data. Hydrological conditions and vertical temperature profiles data in 2010.

## Additional Information and Declarations

### Data Availability

The following information was supplied regarding data availability: The raw data are available in the Supplemental Files.
